# Kidney and cardiovascular-protective benefits of combination drug therapies in chronic kidney disease associated with type 2 diabetes

**DOI:** 10.1186/s12882-024-03652-5

**Published:** 2024-08-01

**Authors:** Muhammad Shahzeb Khan, Janice P. Lea

**Affiliations:** 1 Baylor Research Institute and Baylor Heart Hospital, Dallas, TX USA; 2 Division of Renal Medicine, Department of Internal Medicine, Emory School of Medicine, Atlanta, GA USA

**Keywords:** Chronic kidney disease, Combination therapy, Finerenone, Sodium-glucose cotransporter-2 inhibitor, Glucagon-like peptide-1 receptor agonist

## Abstract

Given the substantial burden of chronic kidney disease associated with type 2 diabetes, an aggressive approach to treatment is required. Despite the benefits of guideline-directed therapy, there remains a high residual risk of continuing progression of chronic kidney disease and of cardiovascular events. Historically, a linear approach to pharmacologic management of chronic kidney disease has been used, in which drugs are added, then adjusted, optimized, or stopped in a stepwise manner based on their efficacy, toxicity, effects on a patient’s quality of life, and cost. However, there are disadvantages to this approach, which may result in missing a window of opportunity to slow chronic kidney disease progression. Instead, a pillar approach has been proposed to enable earlier treatment that simultaneously targets multiple pathways involved in disease progression. Combination therapy in patients with chronic kidney disease associated with type 2 diabetes is being investigated in several clinical trials. In this article, we discuss current treatment options for patients with chronic kidney disease associated with type 2 diabetes and provide a rationale for tailored combinations of therapies with complementary mechanisms of action to optimize therapy using a pillar-based treatment strategy. [This article includes a plain language summary as an additional file].

**Supplementary Information:**

The online version contains supplementary material available at 10.1186/s12882-024-03652-5.

## Introduction

Type 2 diabetes (T2D) is the leading cause of end-stage kidney disease (ESKD) globally [1]. In the United States between 2017 and 2020, approximately one in three people with T2D had chronic kidney disease (CKD) [2]. The presence of both T2D and CKD poses a major public health challenge because patients are at an increased risk of cardiovascular (CV) events and related mortality compared with patients with either condition alone [3]. For example, results from an observational cohort study involving 3211 people from the Jackson Heart Study showed that the combination of T2D and CKD was associated with a greater excess risk for incident stroke, coronary heart disease, and CV mortality versus those with T2D or CKD alone [4]. 

The global burden of CKD resulting from T2D has increased substantially over the last three decades [5]. While CKD was the 19th leading cause of death globally in 1990, it is now the 11th leading cause of death, due to an increasing incidence of T2D and hypertension that together contribute to > 50% of deaths in patients with CKD [6]. The burden of CKD was demonstrated by 2017 data, which showed that CKD associated with T2D accounted for one-third of all disability-adjusted life years globally [6]. Having both these conditions is also associated with a considerable economic burden, particularly in those in the moderate/high-risk categories [7]. Medicare reported spending more than $135 billion on CKD in 2020, with greater rates of spending for patients with CKD plus comorbid T2D or heart failure [8, 9]. 

Given the substantial burden of CKD associated with T2D, an aggressive approach to treatment is required [10]. Despite the benefits of guideline-directed therapy, there remains a risk of continuing progression of CKD and of CV events [11]. Newer drug therapies or treatment approaches seek to reduce this (residual) risk. Two important drug classes used in CKD associated with T2D are sodium-glucose cotransporter-2 inhibitors (SGLT2is) and the nonsteroidal mineralocorticoid receptor antagonist (ns-MRA) finerenone. Although both classes of drug have shown significant benefit versus placebo control in phase 3 clinical trials, the residual risk remains high after treatment with either of these drug classes in CKD and T2D (Table 1) [12–16]. For example, in the CREDENCE trial (Canagliflozin and Renal Events in Diabetes with Established Nephropathy Clinical Evaluation trial), the residual risk of the primary composite outcome event of ESKD, doubling of serum creatinine, or renal or CV death with canagliflozin was 11.1% [11, 14]. In the FIDELIO-DKD trial (Finerenone in Reducing Kidney Failure and Disease Progression in Diabetic Kidney Disease trial), the residual risk of the primary composite end point of kidney failure, a sustained decrease of ≤ 40% in estimated glomerular filtration rate (eGFR) from baseline, or death from renal causes with finerenone was 17.8% [11, 12]. Similarly, in the FIGARO-DKD trial (Finerenone in Reducing Cardiovascular Mortality and Morbidity in Diabetic Kidney Disease trial), the residual risk of the primary composite end point of death from CV causes, nonfatal myocardial infarction, nonfatal stroke, or hospitalization for heart failure was 12.4% [11, 13]. Data such as these highlight a need for novel treatment approaches that may include combining drugs of different classes to reduce residual risk.

The aim of this article is to review current treatment options for patients with CKD associated with T2D in the United States and provide the rationale for tailored combinations of therapies with complementary mechanisms of action (MOAs) to optimize therapy using a pillar-based treatment strategy.

**Table 1:** Residual risk^*^ of kidney/CV events from phase-3 trials of drugs treatments for CKD and T2D

Trial ID	Treatment Arm	Outcome Measures	Residual Risk	
ns-MRA				
FIDELIO-DKD [12]	Finerenone (*N* = 2833)	Kidney failure, a sustained decrease in eGFR of ≥ 40% from baseline, or death from renal causes (primary composite outcome) Death from CV causes, nonfatal myocardial infarction, nonfatal stroke, or hospitalization for heart failure (secondary composite outcome)	17.8% (*n* = 504) 13.0% (*n* = 367)	
		Death from CV causes (outcome event)	4.5% (*n* = 128)	
		Hospitalization for heart failure (outcome event)	4.9% (*n* = 139)	
FIGARO-DKD [13]	Finerenone (*N* = 3686)	Death from CV causes, nonfatal myocardial infarction, nonfatal stroke, or hospitalization for heart failure (primary composite outcome) Death from CV causes (outcome event)	12.4% (*n* = 458) 5.3% (*n* = 194)	
		Hospitalization for heart failure (outcome event)	3.2% (*n* = 117)	
		Kidney failure, a sustained decrease in eGFR of ≥ 40% from baseline, or death from renal causes (secondary composite outcome)	9.5% (*n* = 350)	
SGLT2i				
CREDENCE [14]	Canaglifozin (*N* = 2202)	ESKD, doubling of serum creatinine, or renal or CV death (primary composite outcome)	11.1% (*n* = 245)	
		CV death or hospitalization for heart failure (secondary outcome)	8.1% (*n* = 179)	
		Hospitalization for heart failure (secondary outcome)	4.0% (*n* = 89)	
DAPA-CKD [15]	Dapaglifozin (*N* = 2152)	Sustained decrease in eGFR of ≥ 50% from baseline, ESKD, or death from renal or CV causes (primary composite outcome)	9.2% (*n* = 197)	
		Composite of death from CV causes or hospitalization for heart failure (secondary outcome)	4.6% (*n* = 100)	
EMPA-KIDNEY [16]	Empagliflozin (*N* = 3304)	Composite of progression of kidney disease or death from CV causes (primary outcome)	13.1% (*n* = 432)	
		Hospitalization for heart failure or death from CV causes (secondary outcome)	4.0% (*n* = 131)	

CV, cardiovascular; ESKD, end-stage kidney disease; eGFR, estimated glomerular filtration rate; ns-MRA, nonsteroidal mineralocorticoid receptor antagonist; SGLT2i, sodium-glucose cotransporter-2 inhibitor. ^*^Residual risk is the proportion of patients who received the test drug but their disease progressed and/or they had a kidney or cardiovascular event; for these patients the test drug did not meet the main study efficacy end points (based on calculations included in the paper by Chaudhuri et al [11])

## Treatment of CKD associated with T2D

CKD associated with T2D is a complex disease with multifactorial pathophysiology [17]. In addition to lifestyle management, CKD may be treated with a range of drugs with differing MOAs and clinical effects [6, 18–20]. 

**Fig. 1:**
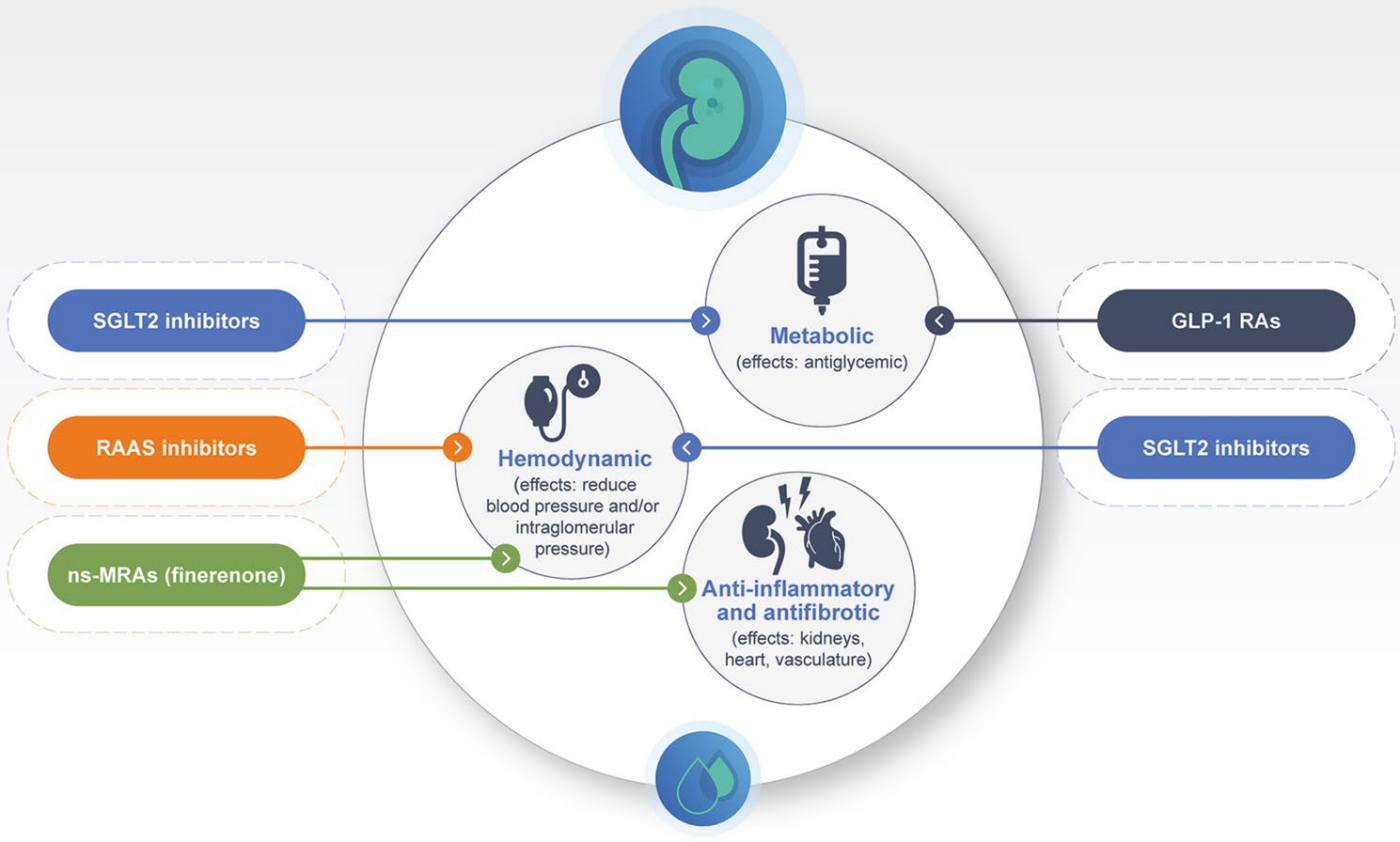
Overview of the MOAs of the main drug classes used in CKD associated with T2D. The figure shows how these main drug classes tackle the three main contributing pathways of CKD. These drugs may be used in combination depending on factors such as baseline clinical factors/treatment aims, patient preference and clinician familiarity [12, 18–20, 32, 33]. GLP-1 RA, glucagon-like peptide-1 receptor agonist; RAAS, renin-angiotensin‐aldosterone system; ns-MRA, nonsteroidal mineralocorticoid receptor antagonist; SGLT2, sodium-glucose cotransporter-2

Drug classes indicated for use in CKD and T2D in the United States include renin-angiotensin-aldosterone system (RAAS) inhibitors, SGLT2is, and finerenone, and for the treatment of T2D with high CV risk, glucagon-like peptide-1 receptor agonists (GLP-1 RAs). Figure 1 provides an overview of the primary MOAs of RAAS inhibitors, SGLT2is, GLP-1 RAs, and finerenone in CKD associated with T2D. The two main RAAS inhibitor subclasses are angiotensin-converting enzyme inhibitors (ACEis) and angiotensin receptor blockers (ARBs), which have been long established as blood pressure-lowering medications [19]. Three SGLT2is—dapagliflozin, canagliflozin, and empagliflozin—are approved for the reduction in kidney disease progression and CV death [21–23]. Three GLP-1 RAs—liraglutide, semaglutide, and dulaglutide—are approved for use in patients with T2D and established CV disease, and a fourth is under FDA review [24–27]. Recent results from the placebo-controlled phase 3 FLOW trial showed that semaglutide treatment significantly reduced the risk of clinically important kidney outcomes and death from CV causes in patients with CKD associated with T2D [28] (Table 2), thus, representing a potential additional future treatment option for patients with CKD associated with T2D. Finerenone is currently the only FDA-approved ns-MRA for the reduction in kidney disease progression and CV death in patients with T2D [29]. Finerenone is also being investigated in a phase 3 placebo-controlled study in non-diabetic CKD (FIND-CKD) (Table 3). Two further drug classes are being investigated for their potential use in CKD and diabetes. These are the aldosterone synthase inhibitor BI 690517, which has just completed phase 2 evaluation and was tested in combination with a RAAS inhibitor and a SGLT2i (empagliflozin) [30], and is now planned for phase 3 testing in the same target patient population, and the endothelin receptor antagonist atrasentan, which is being evaluated for potential application in CKD and diabetes [31]. 

**Table 2:** Published phase-3 data of drug treatments for CKD and T2D and for T2D-patients at high CV risk

Trial ID	Population	Treatment Arms	Median Follow-up	Primary End Point	AEs of Special Interest
ns-MRA					
FIDELIO-DKD [12]	T2D and CKD treated with an ACEi/ARB (max dose without unacceptable side effects)	Finerenone + ACEi/ARB (*N* = 2833) vs. Placebo + ACEi/ARB (*N* = 2841)	2.6 y	Time to kidney failure, a sustained decrease of ≥40% in eGFR from baseline over a period of ≥4 weeks, or death from renal causes 17.8% vs. 21.1% (HR 0.82 [95% CI: 0.73, 0.93], *P* = 0.001)	Hyperkalemia: 18.3% vs. 9.0% Serious hyperkalemia: 1.6% vs. 0.4% K^+^ >5.5 mmol/L: 21.7% vs. 4.5% K^+^ >6 mmol/L: 9.8% vs. 1.4%
FIDELIO-DKD [34] subgroup analyses*	–	Finerenone + ACEi/ ARB + SGLT2i at BL (*N* = 124) & Finerenone + ACEi/ARB (*N* = 2703)	–	HR 1.38 (95% CI: 0.61, 3.10) vs. HR 0.82 (95% CI: 0.72, 0.92); *P*_interaction_= 0.21 (no significant difference in subgroups)	Hyperkalemia: 4.0% vs. 12.1% K^+^ >5.5 mmol/L: 0.0% vs. 4.7% K^+^ >6 mmol/L: 6.5% vs. 21.8%
FIDELIO-DKD subgroup analysis [35]	–	Finerenone + ACEi/ARB + GLP-1 RA at BL (*n* = 189) & Finerenone + ACEi/ARB (*n* = 2644)	–	HR 1.17 (95% CI: 0.71, 1.90) vs. HR 0.80 (95% CI: 0.71, 0.91); *P*_interaction_ = 0.15 (no significant difference in subgroups)	Hyperkalemia: 12.7% vs. 11.7% Serious hyperkalemia: 1.1% vs. 0.9% (related to study drug)
FIGARO-DKD [13]	T2D and CKD treated with an ACEi/ARB (max dose without unacceptable side effects)	Finerenone + ACEi/ARB (*N* = 3686) vs. Placebo + ACEi/ARB (*N* = 3666)	3.4 y	Time to death from CV causes, nonfatal MI, nonfatal stroke, or hospitalization for heart failure 12.4% vs. 14.2% (HR 0.87 [95% CI: 0.76, 0.98], *P* = 0.03)	Hyperkalemia: 10.8% vs. 5.3% Serious hyperkalemia: 0.7% vs. 0.1%
** SGLT2i**					
CREDENCE [14]	T2D and CKD treated with an ACEi/ARB at a stable dose for 4 weeks	Canagliflozin + ACEi/ARB (*N* = 2202) vs. Placebo + ACEi/ARB (*N* = 2199)	2.6 y	Rate of ESKD, doubling of the sCR level from baseline sustained for 30 days, or death from renal or CV causes 43.2 vs. 61.2 per 1000 PY (HR 0.70 [95% CI: 0.59, 0.82], *P* = 0.00001)	Hyperkalemia: 6.9% vs. 8.2% Serious hyperkalemia: NR DKA: 0.5% vs. < 0.1%
DAPA-CKD [15]	CKD with/without T2D treated with an ACEi/ARB at a stable dose for 4 weeks^‡^	Dapagliflozin + ACEi/ARB (*N* = 2152) vs. Placebo + ACEi/ARB (*N* = 2152)	2.4 y	Time to (first occurrence) decline of ≥50% eGFR, onset of ESKD, or death from renal or CV causes 9.2% vs. 14.5% (HR 0.61 [95% CI: 0.51, 0.72], *P* < 0.001)	Hyperkalemia: NR Serious hyperkalemia: NR DKA: 0.0% vs. < 0.1%
EMPA-KIDNEY [16]	CKD with an ACEi^†‡,^ at a clinically appropriate dose	Empagliflozin + ACEi/ARB (*N* = 3304) vs. Placebo + ACEi/ARB (*N* = 3305)	2.0 y	First occurrence kidney disease progression (onset of ESKD) or death from CV disease 13.1% vs. 16.9% (HR 0.72 [95% CI: 0.64, 0.82], *P* < 0.001)	Hyperkalemia: NR Serious hyperkalemia: 2.8% vs. 3.3% Ketoacidosis: 0.2% vs. < 0.1%
GLP-1 RA					
LEADER [36]	T2D with established CVD/CKD if aged 50 y or with high CV risk if aged 60 y	Liraglutide + SOC (*N* = 4668) vs. Placebo + SOC (*N* = 4672)	3.5 y	Time to (first occurrence) death from CV causes, nonfatal (including silent) MI, or nonfatal stroke 13.0% vs. 14.9% (HR 0.87 [95% CI: 0.78, 0.97], *P* = 0.01)	Hyperkalemia: NR Serious hyperkalemia: NR Severe hypoglycemia: 2.4% vs. 3.3%
SUSTAIN-6 [37]	T2D with established CVD/CKD if aged 50 y or with high CV risk if aged 60 y	Semaglutide + SOC (*N* = 1648) vs. Placebo + SOC (*N* = 1649)	2.1 y	First occurrence of death from CV causes, nonfatal (including silent) MI, or nonfatal stroke 6.6% vs. 8.9% (HR 0.74 [95% CI: 0.58, 0.95], *P* = 0.02 for superiority)	Hyperkalemia: NR Serious hyperkalemia: NR Hypoglycemia (0.5 and 1.0 mg doses: semaglutide vs. placebo): 23.1% and 21.7% vs. 21.5% and 21.0%
REWIND [38]	T2D with CVD if aged 50 y, or CVD/CKD if 55 y, or with multiple CV risk factors if aged 60 y	Dulaglutide + SOC (*N* = 4949) vs. Placebo + SOC (*N* = 4952)	5.4 y	First occurrence of nonfatal (including silent) MI, nonfatal stroke, or death from CV or unknown causes 12.0% vs. 13.4% (HR 0.88 [95% CI: 0.79, 0.99], *P* = 0.026)	Hyperkalemia: NR Serious hyperkalemia: NR Severe hypoglycemia: 1.3% vs. 1.5%
AMPLITUDE-O [27]	T2D with history of CVD, or CKD plus one additional CV risk factor if aged 50/55 y (M/F)	Efpeglenatide + SOC (*N* = 2717) vs. Placebo + SOC (*N* = 1359)	1.8 y	First occurrence of nonfatal MI, nonfatal stroke, or death from CV or unknown causes 7.0% vs. 9.2% (HR 0.73 [95% CI: 0.58, 0.92], *P* = 0.007 for superiority)	Hyperkalemia: NR Serious hyperkalemia: NR Severe hypoglycemia: 0.9% vs. 1.0%
FLOW [28]	T2D with high-risk CKD treated with stable maximum tolerated dose of a ACEi or ARB	Semaglutide + SOC (*N* = 1767) vs. Placebo + SOC (*N* = 1766)	3.4 y	The primary outcome event (major kidney disease events [composite of kidney failure, a sustained (for ≥ 28 days) 50% or greater reduction in eGFR from baseline, or death from kidney-related or CV causes]) occurred less frequently with semaglutide vs. placebo 331 first events [5.8 per 100 patient-years of follow-up] vs. 410 first events [7.5 per 100 patient-years]), resulting in a 24% lower RR of the primary outcome in the semaglutide group (HR 0.76 [95% CI: 0.66 to 0.88], *P* = 0.0003)	Hyperkalemia: NR Serious hyperkalemia: NR Severe hypoglycemia: 2.1% vs. 2.1%

ACEi, angiotensin-converting enzyme inhibitor; AEs, adverse events; ARB, angiotensin receptor blocker; BL, baseline; CI, confidence interval; CKD, chronic kidney disease; CV, cardiovascular; DKA, diabetic ketoacidosis; eGFR, estimated glomerular filtration rate; ESKD, end-stage kidney disease; F, female; GLP-1 RA, glucagon-like peptide-1 receptor agonist; HR, hazard ratio; K^+^, potassium levels; M, male; MI, myocardial infarction; NR, not reported; ns-MRA, nonsteroidal mineralocorticoid receptor antagonist; PY, patient-years; RR, relative risk; sCR, serum creatinine; SGLT2i, sodium-glucose cotransporter-2 inhibitor; SOC, standard of care; T2D, type 2 diabetes; y, years. ^*^As per the primary analysis unless otherwise stated. ^†^Patients with and without T2D were permitted to participate (> 96% of patients had T2D at baseline). ^‡^Patients unable to take ACEis/ARBs were allowed to participate

**Table 3:** Ongoing and recently completed clinical trials of three MOA-class combinations* under investigation for CKD

Trial ID	Phase	Population (estimated enrollment)	Treatment Arms	Primary Endpoint	Study Start Date	Estimated Primary Completion
CONFIDENCE [NCT05254002]	2	T2D and CKD treated with an ACEi/ARB (max dose without unacceptable side effects) (*N* ~ 807)	Finerenone + ACEi/ARB + empagliflozin vs. Finerenone + ACEi/ARB vs. Empagliflozin + ACEi/ARB	ΔUACR at 180 days for combination vs. finerenone or empagliflozin alone	June 23, 2023	January 31, 2025
FLAMINGO [NCT05640180]	Observational	T2D and CKD treated with an ACEi/ARB (max dose without unacceptable side effects) SGLT2i use at BL Patients from FIDELIO-DKD and FIGARO-DKD phase 3 trials (*N* ~ 30,000)	Finerenone + ACEi/ARB + SGLT2i vs. Placebo + ACEi/ARB + SGLT2i	Time to kidney failure, a sustained decrease of ≥40% in eGFR from BL over a period of ≥4 weeks or death from renal causes Time to death from CV causes, nonfatal MI, nonfatal stroke, or hospitalization for heart failure	November 23, 2022	Study completed (December 20, 2023)
FIND-CKD [NCT05047263]	3	Non-diabetic CKD treated with an ACEi/ARB (max dose without unacceptable side effects) (*N* ~ 1580)	Finerenone + ACEi/ARB vs. Placebo + ACEi/ARB	Mean rate of change as measured by the total slope of eGFR from BL to Month 32	September 21, 2021	January 12, 2026
MIRACLE [NCT04595370]	2b	Heart failure with LVEF < 60% and CKD; stable background treatment for heart failure, hypertension, T2D or renal disease (*N* ~ 153)	Balcinrenone^†^ (3 different doses) + dapagliflozin vs. Dapagliflozin	% ΔUACR at 12 weeks combination vs. dapagliflozin alone	January 26, 2021	Study completed (September 26, 2023)
NCT05182840	2	CKD (with or without diabetes) treated with an ACEi/ARB (clinically appropriate dose and without unacceptable side effects) (*N* ~ 552)	BI 690517 vs. placebo to BI 690517 vs. Empagliflozin vs. placebo to empagliflozin	%ΔUACR measured in first morning void urine from BL (second randomization) to the end of treatment	January 11, 2022	June 19, 2023
EASi-KIDNEY Source: https://www.ctsu.ox.ac.uk/research/easi-kidney (trial details not yet accessible via clinicaltrials.gov or EU clinical trials register)	3	CKD (with or without diabetes) (*N* ~ 11,000)	BI 690517 + SOC + empagliflozin vs. placebo + SOC + empagliflozin	Risk of kidney disease progression hospitalization for heart failure or death from CV causes Source: https://www.ctsu.ox.ac.uk/research/easi-kidney	~ 2024	~ 2028
FLOW [NCT03819153]	3	T2D and CKD treated with an ACEi/ARB (max dose without unacceptable side effects) (*N* ~ 3508)	Semaglutide + ACEi/ARB vs. Placebo + ACEi/ARB	Time to (first occurrence) decline of ≥50% eGFR, onset of ESKD, or death from kidney disease or CV disease	June 17, 2019	Study completed early for efficacy (interim analysis)
REMODEL [NCT04865770]	3	T2D and CKD treated with an ACEi/ARB (max dose without unacceptable side effects) (*N* ~ 105)	Semaglutide + ACEi/ARB vs. Placebo + ACEi/ARB	ΔKidney oxygenation (cortex), BOLD MRI ΔKidney oxygenation (medulla), BOLD MRI (R2) ΔGlobal kidney oxygenation perfusion (MRI) ΔKidney inflammation (cortex), T1 mapping (MRI) ΔKidney inflammation (medulla), T1 mapping (MRI)	April 28, 2021	October 7, 2024

*ns-MRA, SGLT2i, and/or GLP-1 RA; ^†^Also known as AZD9977. Further details for all studies included in the table can be found at https://clinicaltrials.gov/. ACEi, angiotensin-converting enzyme inhibitor; ARB, angiotensin receptor blocker; BL, baseline; BOLD, blood oxygenation-level dependent; CKD, chronic kidney disease; CV, cardiovascular; eGFR, estimated glomerular filtration rate; ESKD, end-stage kidney disease; GLP-1 RA, glucagon-like peptide-1 receptor agonist; LVEF, left ventricular ejection fraction; MI, myocardial infarction; MRI, magnetic resonance imaging; SGLT2i, sodium-glucose cotransporter-2 inhibitor; T2D, type 2 diabetes; UACR, urine albumin-to-creatine ratio

## Different approaches in the management of CKD associated with T2D

**Fig. 2:**
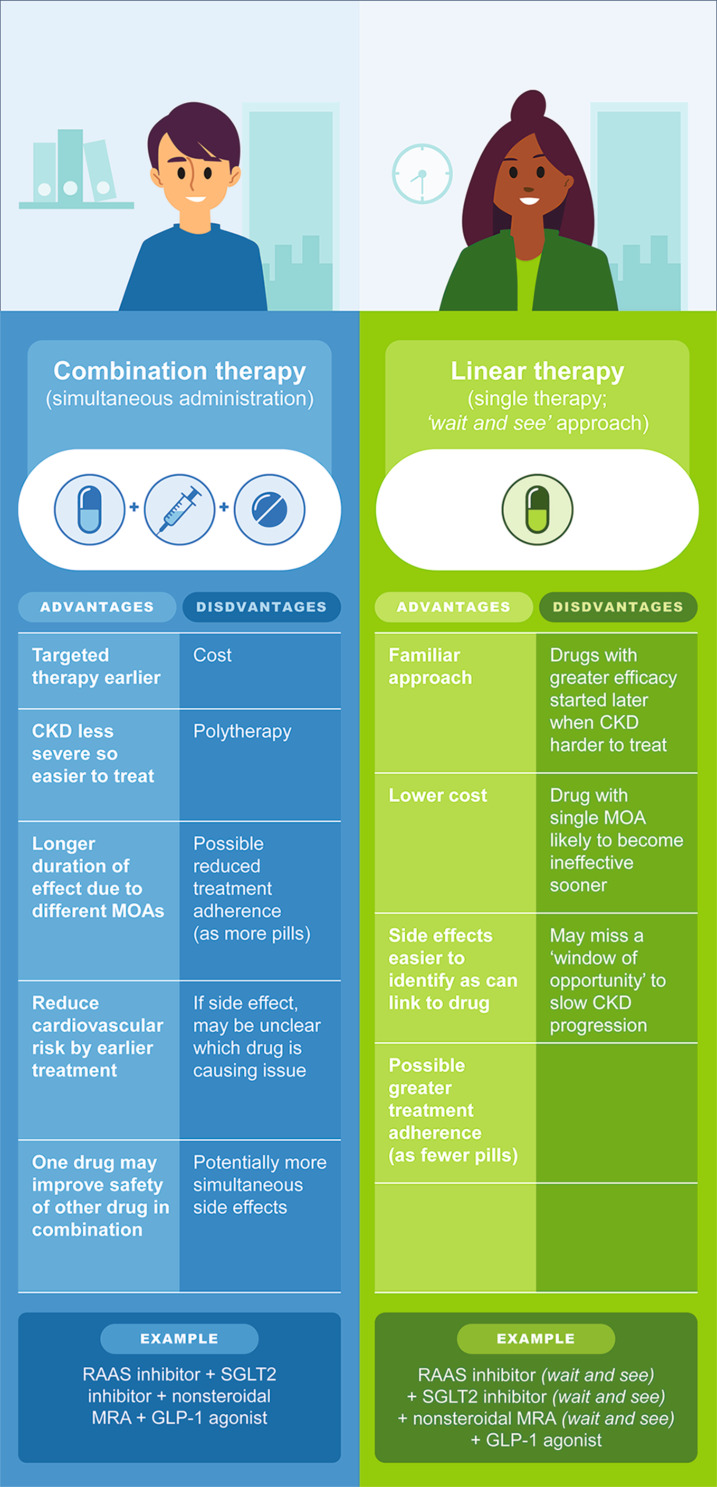
Summary of the advantages and disadvantages of combination (pillar-based) therapy compared with linear therapy approaches in CKD associated with T2D

Historically, a linear approach to pharmacologic management has been used, in which drugs are added then adjusted, optimized, or stopped in a stepwise manner based on their efficacy, toxicity, effects on a patient’s quality of life, and cost [39]. (Fig. 2) However, there are several disadvantages to this approach, which may result in missing a window of opportunity to slow CKD progression. For example, the linear approach may involve delays while assessing if a particular treatment is effective and evaluating factors, such as risk of progression to CKD and other comorbidities, before moving on to the next therapy. Furthermore, the progressive nature of CKD and T2D means that specific first-line therapies may only be useful for short periods, for instance, if glycemic control is lost or if residual symptoms limit their use.

Regular biomarker assessment can enable clinicians to monitor and track CKD progression. A useful biomarker for assessing kidney damage is the urine albumin-creatinine ratio (UACR). A UACR value > 30 mg/g on repeat testing suggests that there is kidney damage and, in this population, the treatment target to slow CKD progression is a ≥ 30% reduction in UACR. [20, 39] The eGFR (or serum creatinine) is a useful biomarker for assessing kidney function and the UACR and eGFR should be measured together, particularly for staging CKD [6, 18]. Failure to achieve UACR targets suggests that the treatment is insufficient at slowing CKD progression, so combination with a drug with a different MOA or switching therapy is recommended. However, it is important to acknowledge that both the UACR and eGFR are surrogate markers for treatment effects and consequently have some inherent limitations when extrapolating to the clinical situation. Values for both markers will be influenced by factors such as intrasubject day-to-day variation (which is why repeat confirmatory testing is recommended by treatment guideline developers), acute drug effects (such as the initial eGFR dip following administration of a RAAS inhibitor, for example [40]), different quantification methods resulting in errors in CKD staging [41] and patient age [42]. Although changes in UACR and eGFR are important parameters for testing treatment effect in CKD associated with T2D, they should be used in addition to other clinical measures.

A pillar-based (combination-based) approach is an established treatment pathway in heart failure. This approach is included as a recommended treatment approach for heart failure with reduced ejection fraction (HFrEF) in the AHA/ACC/HFSA (American Heart Association, American College of Cardiology and Heart Failure Society for America) treatment guidelines [43]. A pillar (combination) approach to T2D treatment has been proposed with the goal of reducing T2D-related complications, including CKD [44]. (Fig. 2) The premise of the pillar approach is to enable early treatment that simultaneously targets multiple pathways involved in disease progression [17, 39]. Additionally, it involves different drugs being started simultaneously at initial (low) doses and then dosed to the maximum tolerated dose as appropriate and as recommended for the condition in question [43]. In CKD, a pillar approach would involve simultaneously targeting pathways of hemodynamic perturbation, metabolic dysregulation, and inflammation [17]. The order by which each drug in the CKD and T2D pillar is added depends on individual patient circumstances, such as their pretreatment UACR, eGFR, any contraindications and comorbidities, but generally RAAS inhibitors are added first followed by a SGLT2i or GLP-1 RA, and finerenone [39]. In any case, treatment of CKD associated with T2D is best managed by a multidisciplinary team.

The pillar approach also has several other advantages over the linear strategy. Early use of drugs that have shown to reduce the risk of CKD progression has the advantage of minimizing the risk of “silent” CKD progression (such as progression in the absence of notable changes in eGFR and/or UACR) [39]. In addition, delays to receiving necessary drugs may be reduced, as there is less need for the between-treatment assessments that are required during a linear approach. Furthermore, use of certain drugs within a pillar approach could have the advantage of improving another drug’s safety profile [39]. For instance, while hyperkalemia risk is increased with MRAs and RAAS inhibitors, [6, 17, 18, 20] addition of an SGLT2i to the combination as part of a pillar approach may decrease the occurrence of hyperkalemia events with MRAs [34, 45]. Although there are potential advantages of combination therapy in CKD associated with T2D, it is important to note that further evidence from prospective studies are needed to ascertain whether a combination therapy approach is overall more beneficial for this high-risk patient population.

It is also important to acknowledge that there are some potential challenges of a pillar approach to CKD management. For example, RAAS inhibitors, SGLT2is and finerenone can cause an initial elevation in serum creatinine, especially in high-risk individuals such as those with T2D [46–48]. Here, it may not be clear which drug is causing the issue. However, an initial dip in eGFR (or increase in serum creatinine) after RAAS inhibitor, SGLT2i, or finerenone initiation is not a reason to discontinue treatment unless there is intolerability or a > 30% increase in serum creatinine [6, 12, 18, 46], additionally, SGLT2i therapy should be continued even if the eGFR falls below 20 ml/min per 1.73 m^2^, unless not tolerated, or kidney replacement therapy is initiated [6, 18]. Both RAAS inhibitors and MRAs (steroidal and nonsteroidal MRAs) can cause hyperkalemia, and this can happen if a RAAS inhibitor is taken alone or simultaneously with an MRA [6, 18, 20, 49]. Another consideration is that, with combination therapy, the number of side effects overall may be higher versus linear or single therapy. However, the potentially greater effect of combination therapy should not prevent its consideration if side effects can be mitigated by dose adjustment and monitoring. For example, the results of a study that used actuarial methods to compare the estimated lifetime CV, kidney and mortality benefits of the combination of an SGLT2i plus a GLP-1 RA plus finerenone versus conventional therapy (a RAAS inhibitor plus traditional risk factor control) found that the triple combination has the potential to afford more relevant gains in CV and kidney event-free and overall survival in patients with CKD and T2D versus conventional therapy alone [50]. However, efficacy and safety data from multicombination drug prospective trials in CKD and T2D is currently lacking, although ongoing trials will address this gap in data [39]. In addition to the potential side-effect profile, prescribing several drugs simultaneously could result in polypharmacy and add extra cost [39]. The direct cost of combination therapy will probably be much higher than the cost of drugs prescribed via a linear or single therapy approach and this will be especially the case where there is inadequate subsidization from government and a lack of health insurance available to individuals [51]. However, the potential longer-term health benefit, and ultimately, the overall cost benefit of combination therapy should not be dismissed and is something payors, formularies and healthcare services should consider more closely. Adherence could also be a greater issue with combination therapy versus a single/linear therapy approach because the more drugs a patient needs to take each day the greater the potential for poor treatment adherence [52]. More broadly, the multidisciplinary team working with the patient should be encouraging self-management, which would include good treatment adherence [53]. 

## Combination therapies in CKD associated with T2D

RAAS inhibitors are recommended for patients with T2D who are at high risk of developing CKD or CKD progression, with or without comorbid hypertension, especially in the presence of albuminuria [6, 18, 20]. One of the challenges with RAAS inhibitor treatment is that aldosterone breakthrough, in which serum aldosterone increases during RAAS blockade, may occur about 1 year after RAAS inhibitor initiation and may be associated with CKD progression [54]. Additionally, the potential for aldosterone breakthrough emphasizes the importance of regular UACR and eGFR monitoring during CKD treatment.

Combining a RAAS inhibitor with a drug or drugs with a different MOA may be an important strategy in the long-term management of CKD. Choice of the drug or drugs to combine with a RAAS inhibitor in CKD may depend on many factors, such as the primary CKD treatment goal (reduce albuminuria and/or additional CV protection), current antiglycemic need, cost, and patient and physician preferences. When considering combinations to use, the potential impact of the drug or drugs on potassium levels should be assessed, as hyperkalemia has been shown to occur in around 10% of patients with CKD receiving RAAS inhibitors in clinical practice [55, 56]. However, it is also important to note that many eligible patients are not prescribed or do not have access to guideline-recommended drug therapies in the first place, which presents another challenge to optimizing treatment for patients with CKD and T2D. For example, results from an observational cohort study that included 52,817 American adults with T2D and incident CKD, showed that many (> 80%) had not received a RAAS inhibitor even within one year of meeting CKD criteria [57]. 

The combination of a RAAS inhibitor with a steroidal MRA (spironolactone or eplerenone) may not be suitable, despite steroidal MRAs demonstrating efficacy in conditions such as HFrEF, resistant hypertension [58–61], hyperaldosteronism (spironolactone), as well as reducing proteinuria/albuminuria [60–63]. The. Key data from these trials are summarized inse drugs have not been rigorously evaluated in CKD and T2D and so are not indicated for use in CKD in the United States. Furthermore, the risk of hyperkalemia has been shown to be elevated with spironolactone, [62, 64] and this also limits its use for combination with RAAS inhibitors. It is important to note that there are some important differences between steroidal and nonsteroidal MRAs, which have been described in the review by Agarwal and colleges, published in 2021 [65]. In the next section, we take a closer look at CKD/T2D drug combinations and potential combinations with finerenone.

## Combinations with finerenone

In the phase 3 FIDELIO-DKD and FIGARO-DKD clinical trials, patients with CKD associated with T2D received either finerenone or placebo on a background of the maximum tolerated dose of a RAAS inhibitor [12, 13, 66]. Other baseline medications in FIDELIO-DKD included an SGLT2i in 4.6% of patients and a GLP-1 RA in 6.9% of patients, while in FIGARO-DKD, 8.4% of patients received an SGLT2i and 7.5% received a GLP-1 RA [12, 13]. These studies demonstrated the benefit of a combination (or pillar) approach; patients who received a combination of finerenone and a RAAS inhibitor had significantly improved CV outcomes, as well as a lower risk of CKD progression and CV events compared with those in the placebo group who received RAAS inhibitor alone. Key data from these trials are summarized in Table 2. 

The beneficial effect achieved with RAAS inhibitors and finerenone may be due to both drugs targeting different aspects of the same inflammatory process in the kidneys and CV system, rather than targeting two proximal elements in the RAAS cascade. RAAS inhibitors inhibit the vasoconstrictive activity of aldosterone, which reduces intraglomerular hypertension in the kidneys [67–69]. It is also noteworthy that as well as stimulating release of aldosterone, angiotensin II can also mediate kidney fibrosis independently of aldosterone [70] and may also cause damage to podocytes [71], so RAAS inhibitors are also helpful in this regard. However, residual risk remains after RAAS blockade, [72, 73] which indicates that targeting RAAS alone is insufficient to prevent disease progression. Upregulation of the RAAS in CKD associated with T2D also triggers overactivation of the mineralocorticoid receptor (MR), which is expressed on many cell types [74]. The overactivation of the MR may lead to inflammation and fibrosis in the heart and kidney [74, 75]. Finerenone blocks the pathologic overactivity of the MR and its effects include antifibrotic and anti-inflammatory actions in the kidneys, vasculature, and heart [32, 65, 74, 76, 77]. Therefore, dual RAAS and MR blockade targets the hemodynamic, inflammatory, and fibrotic pathways involved in CKD pathophysiology (Fig. 1).

The small percentage of patients in the FIDELIO-DKD trial who were on triple combination therapy (receiving an SGLT2i on top of finerenone plus maximum tolerated dose RAAS inhibitor) experienced fewer hyperkalemia events. This may be due to SGLT2is protecting against the possible rise in serum potassium levels with finerenone [34]. This regulation of potassium might be related to the increased electronegative charge in the tubules and the moderately increased aldosterone levels observed with SGLT2is, which do not occur with RAAS inhibitors or MRAs [78–81]. Cardiorenal protection was demonstrated in the overall population treated with finerenone (plus RAAS inhibitor at the maximum tolerated dose) in FIDELIO-DKD, and in the small subgroup who received triple combination therapy, efficacy was shown to be independent of inclusion of an SGLT2i in the combination. However, the small sample size somewhat limits the conclusions that can be drawn from this *post hoc* analysis [34]. In addition to these findings, a systematic review and network meta-analysis of data from 36,186 patients with CKD associated with T2D demonstrated a 57% reduction in the risk of hyperkalemia with an SGLT2i added to an MRA compared with MRA alone (relative risk, 0.43 [0.23 to 0.79]) [82]. 

Finerenone is currently underprescribed in clinical practice, which may be due to concerns about possible hyperkalemia. The introduction of novel potassium binders should allay these concerns [83, 84]. However, concerns about risk of hyperkalemia should not be a reason to not prescribe finerenone, as regular monitoring of serum potassium and, if necessary, dose adjustment of finerenone [29] or the RAAS inhibitor should mitigate the risk of hyperkalemia [18, 29]. Furthermore, adding an SGLT2i to combination therapy that includes RAAS inhibitors and finerenone may help to further reduce the risk of hyperkalemia [18, 84]. Another reason for underprescribing of finerenone in clinical practice is lack of coverage by insurers; results of a 2022 analysis of pharmacy claims published by IQVIA showed that the 30-day new patient rejection rate of finerenone for Medicare part D patients was much higher than the rejection rate for commercially insured patients – the impact being potential delays in finerenone coverage [85]. Other reasons for underprescribing of finerenone might be the cost of finerenone versus alternatives, and clinical inertia, which has been observed with SGLT2i and GLP-1 RA uptake [86]. Answers to this speculation on reasons for finerenone underuse may be provided by the ongoing real-world study, FINE-REAL (NCT05348733), which aims to describe treatment patterns in patients with CKD and T2D treated with finerenone in routine clinical practice [87]. 

An additional ongoing study (CONFIDENCE [NCT05254002]) and a recently completed study (FLAMINGO [NCT05640180]) are investigating the effect of adding an SGLT2i to finerenone plus RAAS inhibitor on predefined kidney outcomes (Table 3). Another recently completed study is with the ns-MRA, balcinrenone, which was investigated in combination with the SGLT2i dapagliflozin (phase 2b MIRACLE [NCT04595370] study) (Table 3). These studies should provide further insights into the benefits of combining an SGLT2i with an ns-MRA plus RAAS inhibitor in the management of CKD associated with T2D.

### Combinations with SGLT2is

The key phase 3 clinical trials of SGLT2is in CKD, DAPA-CKD, CREDENCE, and EMPA-KIDNEY, help to inform on the use of certain treatment combinations as most patients were treated with one of these types of drugs also received a RAAS inhibitor (ACEi or ARB) at baseline [14–16]. Key data from the SGLT2i trials in CKD are summarized in Table 2. Each of these trials demonstrated the benefit of combination therapy, whereby combination of an SGLT2i and a RAAS inhibitor reduced the risk of progression or death from CV (or kidney) causes, CV events, and risk of kidney failure compared with RAAS inhibitor/standard of care alone [14–16]. 

The mechanisms involved in the cardiorenal protective effects of SGLT2is in CKD are complex and are not fulling understood but probably include the class effect of decreasing blood pressure, increasing tubuloglomerular feedback, and decreasing plasma volume [88, 89]. The combination of an SGLT2i plus a RAAS inhibitor in CKD is complementary, as RAAS blockade reduces renal injury by reducing high angiotensin II levels, and SGLT2i restores tubuloglomerular balance by blocking glucose reabsorption that helps to reduce intraglomerular pressure and hyperfiltration, thus reducing albuminuria and hypertension [17, 90]. However, SGLT2is are commonly underprescribed for patients who may benefit from their use, such as those at increased risk of cardiorenal disease, [86, 91–93] suggesting that the introduction of specific SGLT2i combinations may be challenging in clinical practice. Studies of SGLT2i plus ns-MRA combinations are ongoing or have recently completed (Table 3).

### Combinations with GLP-1 RAs

Several clinical trials have shown significant CV benefits of GLP-1 RAs in patients with T2D who were at high risk of CV events, with many of these individuals also taking a RAAS inhibitor at baseline [27, 36–38]. In many cases, this benefit of a GLP-1 RA was also seen in patients with or without kidney dysfunction at baseline. Key data from GLP-1 RAs trials are summarized in Table 2.

In T2D, GLP-1–stimulated insulin secretion is diminished, although this process may be restored with administration of a GLP-1 RA [94]. GLP-1 RAs are also associated with gastric emptying and increased satiety, leading to weight loss [95–97]. The importance of this mechanism is highlighted by recent research that suggests that weight loss at thresholds associated with normoglycemia and normotension may reduce CKD [98]. 

GLP-1 RAs and SGLT2is exhibit different mechanisms that affect glucose metabolism; consequently, GLP-1 RAs are typically added to current antiglycemic treatments if there is ongoing hyperglycemia despite treatment and after taking patient factors into account [18, 84]. In some instances, SGLT2is may offer more cardiorenal protection than GLP-1 RAs alone, further indicating a need to combine these therapies to maximize the cardiorenal protection afforded to patients at risk. In a meta-analysis of GLP-1 RA and SGLT2i clinical trials, the authors concluded that the prevention of heart failure and kidney disease progression by SGLT2is should be considered in the decision-making process when treating people with T2D [99]; although SGLT2is may be preferred over GLP-1 RAs in select patients. Overall, combining an SGLT2i and a GLP-1 RA may provide a holistic approach to improving outcomes in patients with CKD and high CV risk [100].

The impact of the GLP-1 RA semaglutide (plus maximum tolerated RAAS inhibitor) on kidney and CV outcomes is being investigated in the ongoing phase 3 FLOW clinical trial in patients with T2D and CKD (NCT0381915) (Table 3). This trial was stopped in October 2023 at interim analysis due to meeting certain prespecified efficacy criteria (Novo Nordisk company announcement, October 10, 2023) (Table 3).

### Guideline recommendations relating to combination therapy in CKD with T2D

**Fig. 3:**
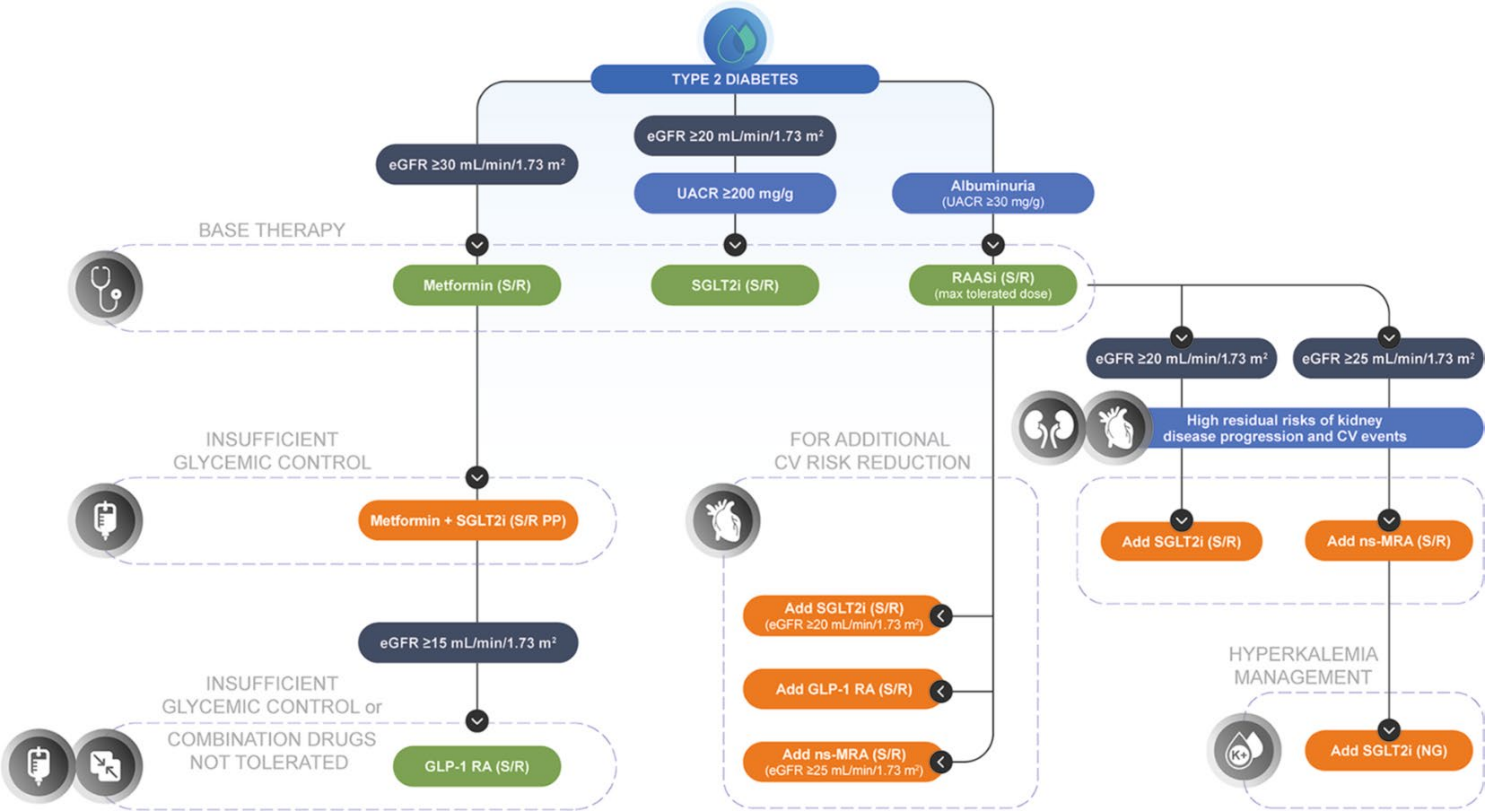
Current practice guideline recommendations for combination therapies in patients with T2D at risk of CKD. KDIGO 2022 [18], ADA 2024 Chap. 11, [20] and AACE 2022 [84] and KDIGO 2024 [6] S/R = strong support/recommended by at least one of the practice guidelines; PP = Practice Point (by KDIGO, not a formal recommendation); NG = not graded AACE, American Association of Clinical Endocrinology; ADA, American Diabetes Association; CKD, chronic kidney disease; CV, cardiovascular; eGFR, estimated glomerular filtration rate; GLP-1 RA, glucagon-like peptide-1 receptor agonist; KDIGO, Kidney Disease: Improving Global Outcomes; ns-MRA, nonsteroidal mineralocorticoid receptor antagonist; SGLT2i, sodium-glucose cotransporter-2 inhibitor; T2D, type 2 diabetes

Three treatment guidelines—Kidney Disease: Improving Global Outcomes (KDIGO) (2022), American Diabetes Association (ADA) (2024), and American Association of Clinical Endocrinology (AACE) (2022)—provide recommendations that relate to drug combinations for specific clinical scenarios in the management of CKD associated with T2D (Fig. 3). The KDIGO also recently published their 2024 update to the Clinical Practice Guideline for the Evaluation and Management of CKD.

Before discussing the specific combinations, the base therapy for the combinations, or first-line drug therapies, needs to be considered. The glucose-lowering agents metformin and SGLT2is are recommended by the KDIGO 2022 guidelines as first-line therapies in patients with T2D, [18] and all three guidelines recommend a RAAS inhibitor, at the maximum tolerated dose, as a standard of care for patients with T2D and albuminuria [18, 20, 84]. 

SGLT2is are recommended if eGFR is ≥20 mL/min/1.73 m^2^ to reduce progression of CKD and risk of CVD, with the KDIGO and ADA guidelines also adding that patients are to have a UACR ≥200 mg/g [6, 18, 20, 84]. Where the combination of metformin and SGLT2i is insufficient to control blood glucose or cannot be tolerated by patients, GLP-1 RAs are recommended [18]; AACE guidelines recommend GLP-1 RAs in patients with eGFR ≥15 mL/min/1.73 m^2^ for glycemic control and to reduce risk of atherosclerotic CV disease and progression of albuminuria [84]. GLP-1 RAs are generally preferred after consideration of several factors when selecting additional drugs to manage glycemia, such as patient preferences, comorbidities, eGFR, and cost [18, 20]. 

None of the guidelines provide clear guidance for use of an SGLT2i and GLP-1 RA combination, although the ADA states that SGLT2i or GLP-1 RA may be considered additionally for CV risk reduction, presumably by adding any of these drugs to a maximum tolerated dose of RAAS inhibitor [20]. KDIGO and ADA treatment guidelines provide a clear recommendation for use of ns-MRA (i.e., finerenone) in combination with a RAAS inhibitor, at a maximum tolerated dose, for patients with T2D and high residual risks of kidney disease progression (UACR ≥30 mg/g [persistent albuminuria]) and CV events, who have an eGFR ≥ 25 mL/min/1.73 m^2^ and normal serum potassium [6, 18, 20]. Finerenone may also be used in combination with an SGLT2i, which is an appropriate strategy to mitigate the hyperkalemia associated with finerenone [6, 18, 84]. Support for the finerenone plus SGLT2i combination (in addition to a RAAS inhibitor) is being investigated in two clinical studies (Table 3).

## Conclusions

Drugs from several drug classes have demonstrated proven efficacy and safety in CKD associated with T2D. However, the residual risk in these patients remains high, which emphasizes a need for novel therapies or novel strategies to facilitate a reduction in residual risk. A pillar approach has been suggested where combinations of drugs with complementary MOAs are used simultaneously rather than sequentially. Inclusion of either an SGLT2i or finerenone to a maximum tolerated dose of a RAAS inhibitor provides supportive phase 3 evidence of a pillar approach. The pillar approach if adopted in routine clinical practice in CKD would enable drugs of different classes to be combined and taken earlier in the disease course, which may have greater benefit as the extent of pathophysiologic damage is likely to be lower at an early CKD stage. Results of ongoing clinical trials investigating different drug class combinations in CKD associated with T2D are awaited with interest. Further guidance will be provided from results of registry studies (such as the ongoing United States Renal Data System [USRDS] registry) that will provide real-world clinical data, which will be more generalizable to the clinical population [This article includes a plain language summary as an additional file].

### Electronic supplementary material

Below is the link to the electronic supplementary material.

Supplementary Material 1

## Data Availability

No datasets were generated or analysed during the current study.

## Abbreviations

ACEis: Angiotensin-converting enzyme inhibitors
ARBs: Angiotensin receptor blockers
CV: Cardiovascular
CKD: Chronic kidney disease
CREDENCE trial: Canagliflozin and Renal Events in Diabetes with Established Nephropathy Clinical Evaluation trial
ESKD: End-stage kidney disease
eGFR: Estimated glomerular filtration rate
FIDELIO-DKD trial: Finerenone in Reducing Kidney Failure and Disease Progression in Diabetic Kidney Disease trial
FIGARO-DKD trial: Finerenone in Reducing Cardiovascular Mortality and Morbidity in Diabetic Kidney Disease trial
GLP-1 RAs: Glucagon-like peptide-1 receptor agonists
MOA: Mechanisms of action
MR: Mineralocorticoid receptor
MRA: Mineralocorticoid receptor antagonist
RAAS inhibitors: Renin angiotensin-aldosterone system inhibitors
SGLT2is: Sodium-glucose cotransporter-2 inhibitors
T2D: Type 2 diabetes
