# Supplementary material for: Kidney and cardiovascular-protective benefits of combination drug therapies in chronic kidney disease associated with type 2 diabetes
Source: BMC Nephrol. 2024 Aug 1;25:248. doi: 10.1186/s12882-024-03652-5 (PMC11293206; doi:10.1186/s12882-024-03652-5)
Supplement: Supplementary file 1 — Supplementary Material 1 [file 12882_2024_3652_MOESM1_ESM.docx]

**Additional File 1. Plain Language Summary**

In this review article, the authors consider the current treatment options available for people living with chronic kidney disease (CKD for short) and type 2 diabetes (T2D for short) in the United States and provide an explanation for using person-specific drug combinations. Many people living with T2D and CKD will take medicines recommended by their healthcare team that keep their blood sugar levels under control (for T2D) and slow the rate at which their CKD worsens over time. Often, patients take one type of drug at a time for each condition they have and then wait to see if it works. They also watch for any side effects (an unwanted reaction to the drug). The amount of the drug (called the dose) may be increased or decreased to find the right balance between achieving the best working result and reducing the risk of getting side effects. However, CKD gets worse over time and this can happen even if the medicine once worked well. When a medicine stops working, a person’s healthcare team may recommend changing to a different medicine that works differently and should again slow how quickly their CKD gets worse. Some healthcare professionals believe that it is better to not wait for a person’s CKD to get worse, when it might be harder to treat, and instead take several medicines that work differently at the same time and sooner. This approach is called combination therapy and is thought it will improve outcomes for people living with CKD and T2D.
